# Morphological and Fractal Properties of Brain Tumors

**DOI:** 10.3389/fphys.2022.878391

**Published:** 2022-06-27

**Authors:** Jacksson Sánchez, Miguel Martín-Landrove

**Affiliations:** ^1^ Faculty of Science and Technology, Physics Department, Universidad Nacional Pedro Henríquez Ureña, Santo Domingo, Dominican Republic; ^2^ Centre for Medical Visualization, National Institute for Bioengineering, INABIO, Universidad Central de Venezuela, Caracas, Venezuela; ^3^ Centro de Diagnóstico Docente Las Mercedes, Caracas, Venezuela

**Keywords:** fractal dimension, scaling analysis, visibility graphs, local roughness exponent, tumor interface, tumor growth dynamics, morphological parameters, tumor surface regularity

## Abstract

Tumor interface dynamics is a complex process determined by cell proliferation and invasion to neighboring tissues. Parameters extracted from the tumor interface fluctuations allow for the characterization of the particular growth model, which could be relevant for an appropriate diagnosis and the correspondent therapeutic strategy. Previous work, based on scaling analysis of the tumor interface, demonstrated that gliomas strictly behave as it is proposed by the Family-Vicsek ansatz, which corresponds to a proliferative-invasive growth model, while for meningiomas and acoustic schwannomas, a proliferative growth model is more suitable. In the present work, other morphological and dynamical descriptors are used as a complementary view, such as surface regularity, one-dimensional fluctuations represented as ordered series and bi-dimensional fluctuations of the tumor interface. These fluctuations were analyzed by Detrended Fluctuation Analysis to determine generalized fractal dimensions. Results indicate that tumor interface fractal dimension, local roughness exponent and surface regularity are parameters that discriminate between gliomas and meningiomas/schwannomas.

## 1 Introduction

Tumor interface exhibits a complex and irregular geometry due to the dynamics involved in the tumor growth process, which in general takes into account tumor cell proliferation and invasion into the surrounding tissue. To characterize its complexity, fractal analysis has been used routinely for tumor detection (Iftekharuddin et al., 2009) and therapy monitoring (Di Ieva et al., 2012). In the case of brain tumors, magnetic resonance imaging techniques give detailed geometrical information with excellent spatial resolution and quality for the evaluation of the tumor interface. Parameters extracted from the tumor interface by scaling and fractal analysis have given relevant clues about the complex tumor growth dynamics (Brú et al., 2012) (Brú et al., 2003) (Brú et al., 2008) (Torres Hoyos and Martín-Landrove, 2012) which in turn can be used to further validate tumor growth models (Brú et al., 2014) for therapy simulation and prognosis. In a previous work (Martín-Landrove et al., 2020) it was demonstrated that scaling analysis provided a clear difference in the tumor growth model for gliomas, which follow a ballistic growth model in completely agreement with the Family-Vicsek ansatz (Family and Vicsek, 1991) (Barabasi and Stanley, 1995), compared to meningiomas/schwannomas. A different approach that includes fractal properties of the tumor interface or surface has been proposed by defining surface regularity measures (Pérez-Beteta et al., 2018) (Popadic et al., 2021). So far, scaling analysis parameters, such as fractal dimension and local roughness exponent, and surface regularity measures give a global picture of the fractal properties of the tumor interface. Since the tumor growth process is heterogeneous it is expected that its fractal properties should be heterogeneous as well and a more general approach is needed and general multifractal analysis methods (Kantelhardt et al., 2002) (Lopes and Betrouni, 2009) (Gu and Zhou, 2006) applied to the fluctuations over the interface have to be used. In the present work, an extended image database is used to determine an extended group of parameters that characterize the tumor interface, including those previously determined (Martín-Landrove et al., 2020).

Related to the assessment of the tumor interface, several methods have been proposed for the segmentation of brain tumors (Işın et al., 2016) (Wadhwa et al., 2019) (Zhao et al., 2020) from magnetic resonance images, which include conventional methods such as thresholding and region growing, supervised methods mainly represented by support vectors machines and artificial neural networks and unsupervised methods which include clustering methods and deformable models. Even though supervised methods perform better than unsupervised ones (Rao et al., 2018), for the purpose of the present work, unsupervised methods, such as K-means or Fuzzy C-means are preferable since these methods do not need any training set and in many cases are simpler in its numerical implementation. In the present work, an unsupervised method based on dynamic quantum clustering (Weinstein and Horn, 2009), (Horn and Gottlieb, 2001) is used for image segmentation to determine the tumor interface.

The article is organized as follows, in Section 2 it is described the selection of images for this study, the segmentation method employed for determination of the tumor interface and the different morphological parameters that describe the tumor interface such as fractal dimension and lacunarity, growth dynamics exponents, regularity measures, complex visibility graphs and parameters derived from multifractal analysis. The results are discussed in Section 3 and the conclusions are presented in Section 4.

## 2 Materials and Methods

### 2.1 Image Selection

Images for high grade gliomas were extracted from different collections in The Cancer Imaging Archive (Chang et al., 2011), (Clark et al., 2013); The Cancer Genome Atlas Low Grade Glioma (TCGA-LGG) data collection (Pedano et al., 2020), the Repository of Molecular Brain Neoplasia Data (REMBRANDT) (Scarpace et al., 2019) for astrocytomas and oligodendrogliomas of grades 2 and 3, and The Cancer Genome Atlas Glioblastoma multiforme [TCGA-GBM] collection (Scarpace et al., 2016) for glioblastoma multiforme. Also, data coming from the RSNA-ASNR-MICCAI Brain Tumor Segmentation (BraTS) Challenge 2021 (Baid et al., 2021) (Menze et al., 2015) (Bakas et al., 2017). For other brain neoplasias, such as meningiomas and acoustic schwannomas, local image datasets were used. Among these collections, only contrast enhanced T1-weighted images, with tumor lesions clearly identified as such and separated from anatomical structures, were selected for analysis.

### 2.2 Clustering of Data and Image Segmentation

Image digital levels were clustered using the Dynamic Quantum Clustering algorithm (DQC) (Weinstein and Horn, 2009) (Horn and Gottlieb, 2001) which assumes that data are described by a set of points, each one defined with some uncertainty, *σ* and the distribution for all points in data space is given by a Parzen estimator, *φ*, which satisfies the time independent Schrödinger equation for its ground state,

$$-\frac{1}{2\sigma^{2}}\nabla^{2}\varphi +V\left(\vec{X}\right)\varphi =E\varphi =0$$ (1)

which leads to the evaluation of an energy potential

$$V\left(\vec{X}\right)=\frac{1}{2\sigma^{2}}\frac{\nabla^{2}\varphi }{\varphi }$$ (2)

**FIGURE 1:**
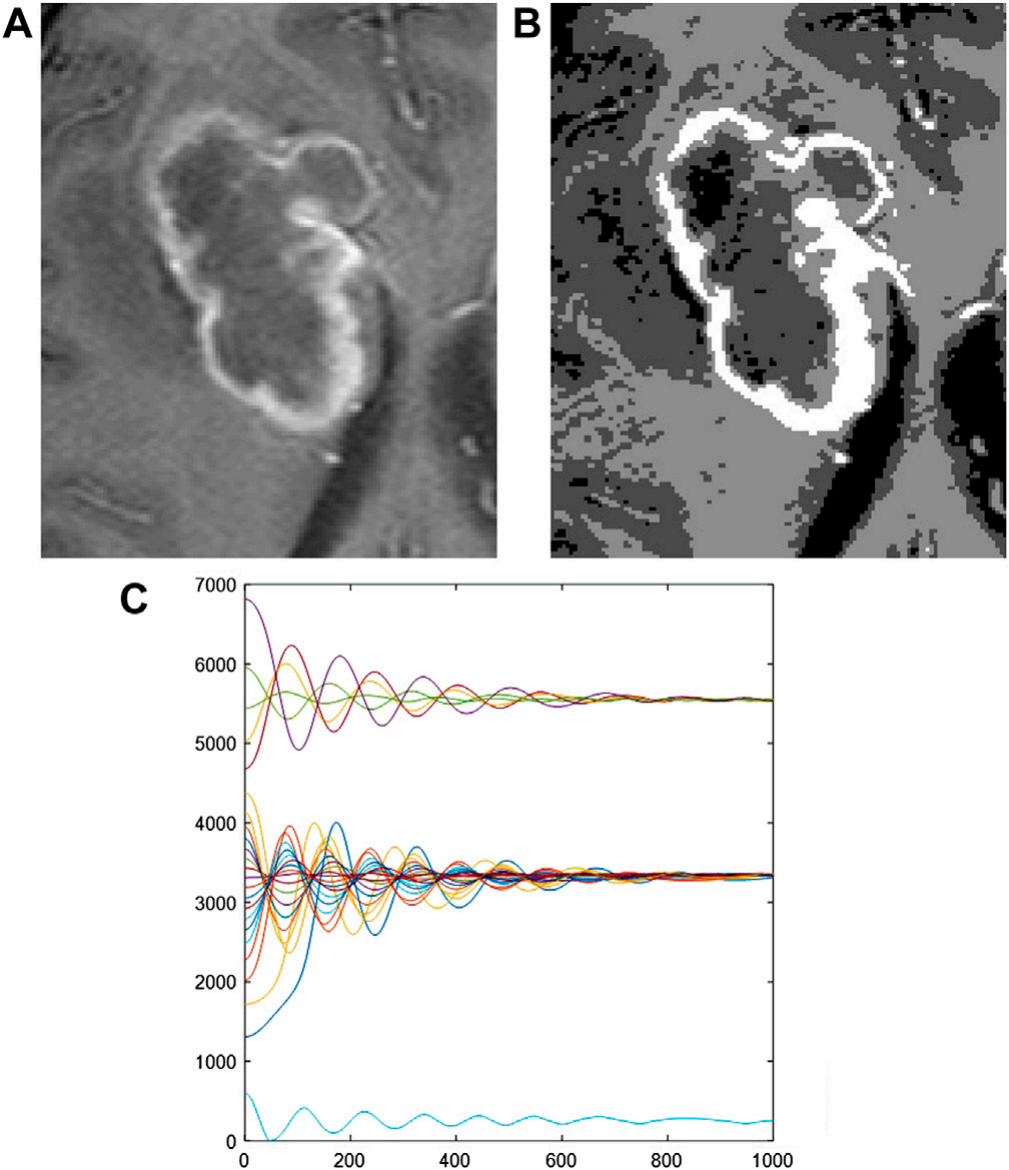
Clustering of digital levels. **(A)** Original image, **(B)** clustered image, **(C)** Time evolution of digital levels.

The number of potential minima (Horn and Gottlieb, 2001) was previously used (Martín-Landrove et al., 2020) to determine the number of classes in a K-means algorithm alone. In the present work, the full dynamic quantum clustering algorithm is used and by Ehrenfest theorem, digital levels evolve in time toward the potential minima according to the following equation of motion (Lafata et al., 2018),

$$\sigma^{2}\frac{d^{2}\vec{X_{k}}}{dt^{2}}=-\nabla V\left(\vec{X_{k}}\right)-\gamma \frac{d\vec{X_{k}}}{dt}$$ (3)

which is a second order Langevin equation with a dissipative term *γ*. The clustering process is performed with a suitable selection of parameters such as *σ* ^2^, which is an equivalent to a “mass”, and determines the number of classes among the digital levels (Horn and Gottlieb, 2001), dissipation, *γ* and time interval. These parameters are estimated according to the magnitude of the digital levels in the image and assess the evolution of all the data to the potential minima. An example of clustering of digital levels is shown in Figure 1. For image segmentation the dynamics is performed as a two step process (Sánchez and Martín-Landrove, 2021):

Dynamic A: First application of the dynamics upon the original image using the potential calculated from the original image histogram. At the end of this step, a Parzen estimator is evaluated using the clustered image histogram, allowing for the calculation of a “trap” potential, which defines the number of classes if a further classification algorithm, such as K-means, is to be used. It has been proved that the dynamic quantum clustering algorithm provides the same set of centroids as the K-means algorithm (Sánchez and Martín-Landrove, 2021).

Dynamic B: Second application of the dynamics upon the original image using the “trap” potential.

**FIGURE 2:**
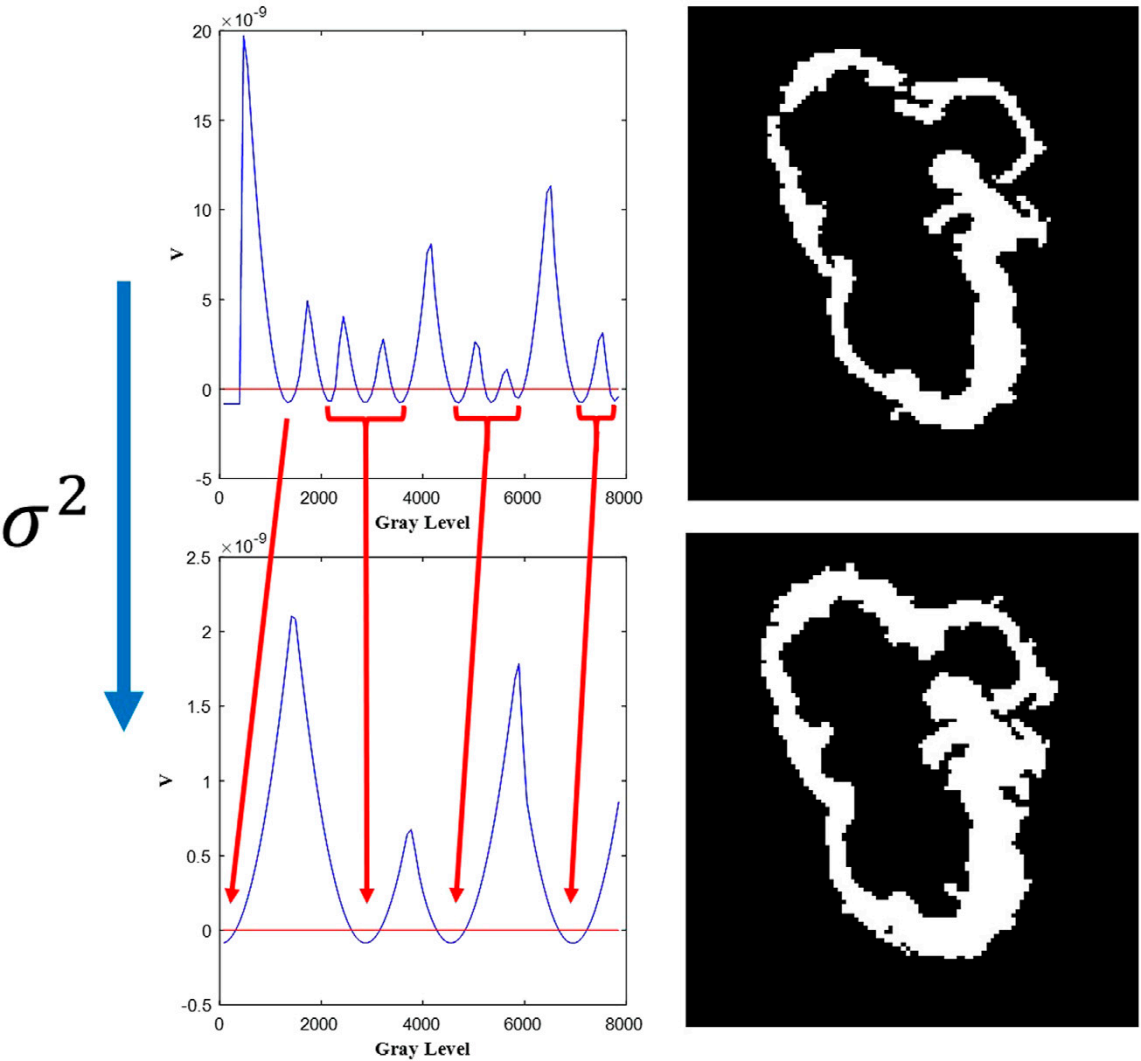
Dependence of the segmentation procedure on the value of the ‘mass’ of the particle, *σ* ^2^. Arrows indicate how the potential energy minima, in the ‘trap’ potential, collapse as *σ* ^2^ is increased from top to bottom by a 3-fold factor. On the right, the corresponding segmentation.

In Figure 2 it is shown how the selection of the appropriate *σ* ^2^ determines the number of classes and therefore the image segmentation. It is important to note that the “trap” potential compared to the original Schrödinger potential, exhibit a well defined minima, which allows for an unsupervised definition of the number of clusters and an improvement in the assessment of the tumor interface.

### 2.3 Morphological Parameters

#### 2.3.1 Fractal Dimension and Lacunarity of the Tumor Interface

Fractal dimension (Iftekharuddin et al., 2003) and lacunarity (Plotnick et al., 1993) have been used as morphological parameters to characterize tumor interface and grading of brain tumors (Smitha et al., 2015) (Park et al., 2020). Both quantities can be calculated using a box counting algorithm and are defined as

$$d_{F}=-\underset{\epsilon \to 0}{\lim}\frac{\log\left(N\left(\epsilon \right)\right)}{\log\left(\epsilon \right)}$$ (4)

with *N* (*ϵ*), the number of boxes containing the fractal object and *ϵ* the size of the box. In a similar way, lacunarity is defined as

$$\lambda \left(\epsilon \right)={\left(\frac{\sigma \left(\epsilon \right)}{\mu \left(\epsilon \right)}\right)}^{2}$$ (5)

where *μ* (*ϵ*) is the mean of point density within the box of size *ϵ* and *σ* (*ϵ*), the standard deviation.

#### 2.3.2 Scaling Analysis and Tumor Growth Dynamics

Tumor interface, in both resected and *in vitro* samples (Brú et al., 2012) (Brú et al., 2003), and *in vivo* (Torres Hoyos and Martín-Landrove, 2012) (Martín-Landrove et al., 2016) (Martín-Landrove et al., 2020) has been characterized using scaling analysis techniques. These studies have shown that tumor contours exhibit super-rough scaling dynamics described by the Family–Vicsek ansatz (Family and Vicsek, 1991) (Barabasi and Stanley, 1995) that corresponds to a ballistic growth process or a proliferative-invasive tumor growth model. The roughness of the tumor interface can be parameterized at the global level, with an exponent *α*, and at the local level by a roughness exponent, *α*_*loc*_. In three dimensions, the local roughness exponent relates the scale-averaged width of the interface between tumor and host to the scale of growth *s*, exhibiting a power-law behavior for small *s* (Torres Hoyos and Martín-Landrove, 2012) (Martín-Landrove et al., 2016):

$$W\left(s\right)∼s^{\alpha_{\mathrm{loc}}}$$ (6)

with *W* given by (Brú et al., 2008),

$$W\left(s,t\right)={\left\{\frac{1}{s}\underset{r_{i}\epsilon s}{\sum }{\left[r_{i}\left(t\right)-{\left\langle r_{i}\right\rangle }_{s}\right]}^{2}\right\}}_{\Sigma }^{\frac{1}{2}}$$ (7)

where ${<r_{i}>}_{s}$ represents the average of the radius, measured from the tumor center, over a patch of scale *s* located at the tumor interface, and ${\left\{*\right\}}_{\Sigma }$ represents the average over all realizations (all possible patches of scale *s*) over the interface surface Σ. In order for the growing process to follow the Family–Vicsek ansatz (Family and Vicsek, 1991), fractal dimension and local roughness exponent are related in a general way (Family and Vicsek, 1991) (Barabasi and Stanley, 1995), i.e., their sum is equal to the embedding dimension of the shape, or Euclidean dimension, *d*_*E*_,

$$\alpha_{\mathrm{loc}}+d_{F}=d_{E}$$ (8)

Also, the saturation value of the interface width, *W*_*sat*_, scales with average of the tumor size, ⟨*R*⟩ as

$$W_{\mathrm{sat}}\left(\langle R\rangle \right)∼{\langle R\rangle }^{\alpha }$$ (9)

where *α* is the global roughness exponent.

#### 2.3.3 Surface Regularity Measures

A surface regularity measure has been proposed to characterize glioblastoma multiforme (Pérez-Beteta et al., 2018) by the following equation,

$$S_{R}=\frac{TV}{TV_{eq}}$$ (10)

where *TV* is the total volume of the tumor and *TV*_*eq*_ is the volume of a sphere which has the same surface area as the tumor, *TS*. Thus

$$S_{R}=6\sqrt{\pi }\frac{TV}{\sqrt{{\left(TS\right)}^{3}}}$$ (11)

In a similar way, a surface factor has been proposed (Popadic et al., 2021) for grading meningioma tumors. the surface factor is defined as,

$$S_{F}=\frac{TS_{eq}}{TS}$$ (12)

where *TS*_*eq*_ is the surface area of an sphere with a volume equal to the tumor volume, *TV* and *TS* is the surface area of the tumor. In terms of *TV* and *TS*, *S*_*F*_ can be written,

$$S_{F}={\left(6\sqrt{\pi }\right)}^{\frac{2}{3}}\frac{TV^{\frac{2}{3}}}{TS}={S_{R}}^{\frac{2}{3}}$$ (13)

So any of the proposed regularity measures *S*_*R*_ or *S*_*F*_ are equivalent for the description of the surface regularity and therefore *S*_*R*_ will be used in the present work. According to (Pérez-Beteta et al., 2018), *S*_*R*_ is related to the fractality or roughness of the tumor surface, i.e., if *S*_*R*_ ≪ 1, tumor exhibits a distinct fractal dimension with a rough surface, while otherwise its fractal dimension is close to the Euclidean one and the tumor surface is smooth. Nevertheless, the actual value of *S*_*R*_ must be corrected by a shape factor due to the fact that the distribution of points over the tumor surface could be elongated along certain directions, departing from the roughed sphere condition,

$${S_{R}}^{*}=\frac{S_{R}}{f_{\mathrm{shape}}}$$ (14)

**FIGURE 3:**
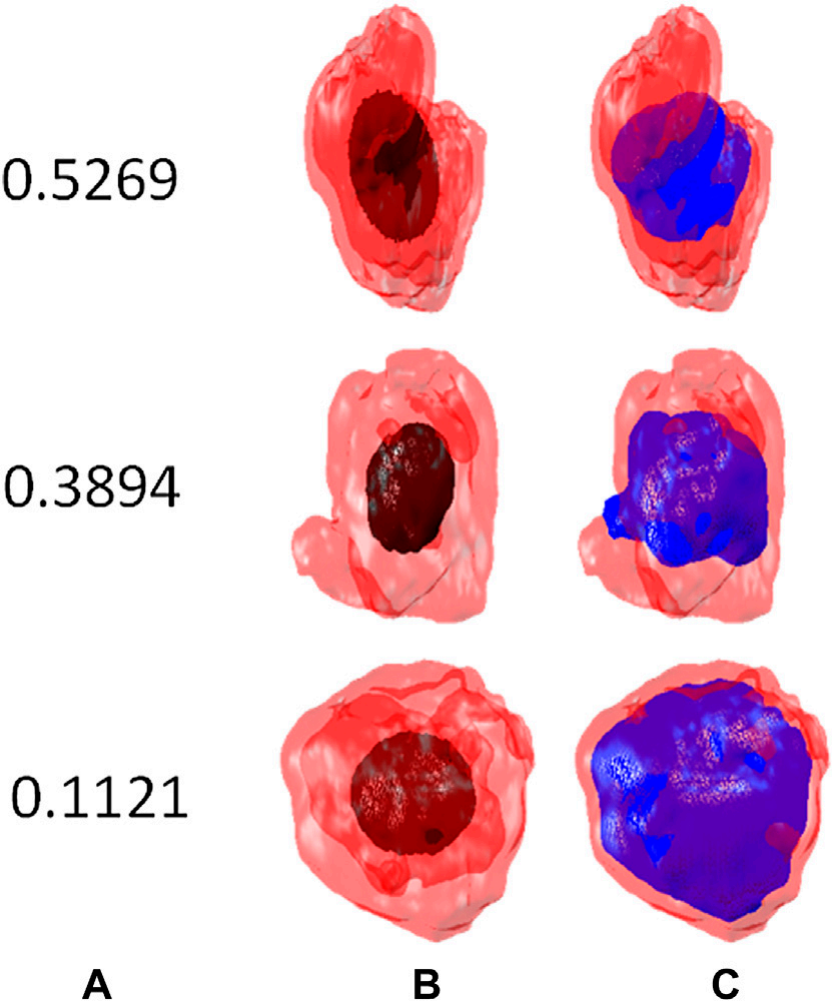
Principal component analysis of the tumor interface. **(A)** fractional anisotropy of the ellipsoid obtained by PCA, **(B)** PCA ellipsoids, (black), located inside the tumor interface (red) and **(C)** tumor interface correction (blue).

*f*_*shape*_ is calculated in a likewise way as *S*_*R*_,

$$f_{\mathrm{shape}}=\frac{V_{\mathrm{ellipsoid}}}{V_{\mathrm{sphere}}}$$ (15)

where *V*_*ellipsoid*_ is the volume of an ellipsoid obtained by principal component analysis of the point distribution over the tumor surface and *V*_*sphere*_ is the volume of a sphere with the same surface area as the ellipsoid. For the tumor interface, the Fractional Anisotropy is defined as,

$$FA={\left\{\frac{{\left(\lambda_{1}-\lambda_{2}\right)}^{2}+{\left(\lambda_{2}-\lambda_{3}\right)}^{2}+{\left(\lambda_{1}-\lambda_{3}\right)}^{2}}{2\left(\lambda_{1}^{2}+\lambda_{2}^{2}+\lambda_{3}^{2}\right)}\right\}}^{\frac{1}{2}}$$ (16)

where *λ*_*i*_, *i* = 1, 2, 3 are the ellipsoid axes. In Figure 3 it is shown how FA determines the tumor interface correction that leads to Equation 14. Another factor can be defined which takes into account the distribution of contrast inside the tumor,

$$S_{C}=\frac{TV_{C}}{TV_{C,eq}}=6\sqrt{\pi }\frac{TV_{C}}{\sqrt{{\left(TS_{C}\right)}^{3}}}$$ (17)

where *TV*_*C*_ is the volume of the region with contrast and *TS*_*C*_ is its total surface area, including inner and outer surfaces.

#### 2.3.4 Ordered Series and Visibility Graphs

Ordered series and the associated visibility graphs (Lacasa et al., 2008) (Lacasa et al., 2009) that can be extracted from the tumor interface has been used to further discriminate between the dynamical models that describe tumor growth (Brú et al., 2014). In a previous work (Martín-Landrove et al., 2020), it has been demonstrated that the associated visibility graph, through its connectivity distribution function *P* (*k*) discriminates between gliomas and meningiomas/schwannomas, in the exponent of its power law behavior,

$$P\left(k\right)∼k^{\gamma }$$ (18)

In the present work, the analysis of the ordered series and its associated graph is extended one step further to determine its scaling properties (Pérez-Beteta et al., 2018). Similarly to Equation 7, the local standard deviation of the vertex degree for a subset *ϕ* on the ordered series can be written as (Brú et al., 2014) (Estrada, 2010),

$$W_{k}\left(\phi \right)={\left\langle \frac{1}{\phi }\underset{k_{i}\epsilon \phi }{\sum }{\left[k_{i}-{\left\langle k_{i}\right\rangle }_{\phi }\right]}^{2}\right\rangle }_{\phi }^{\frac{1}{2}}$$ (19)

**FIGURE 4:**
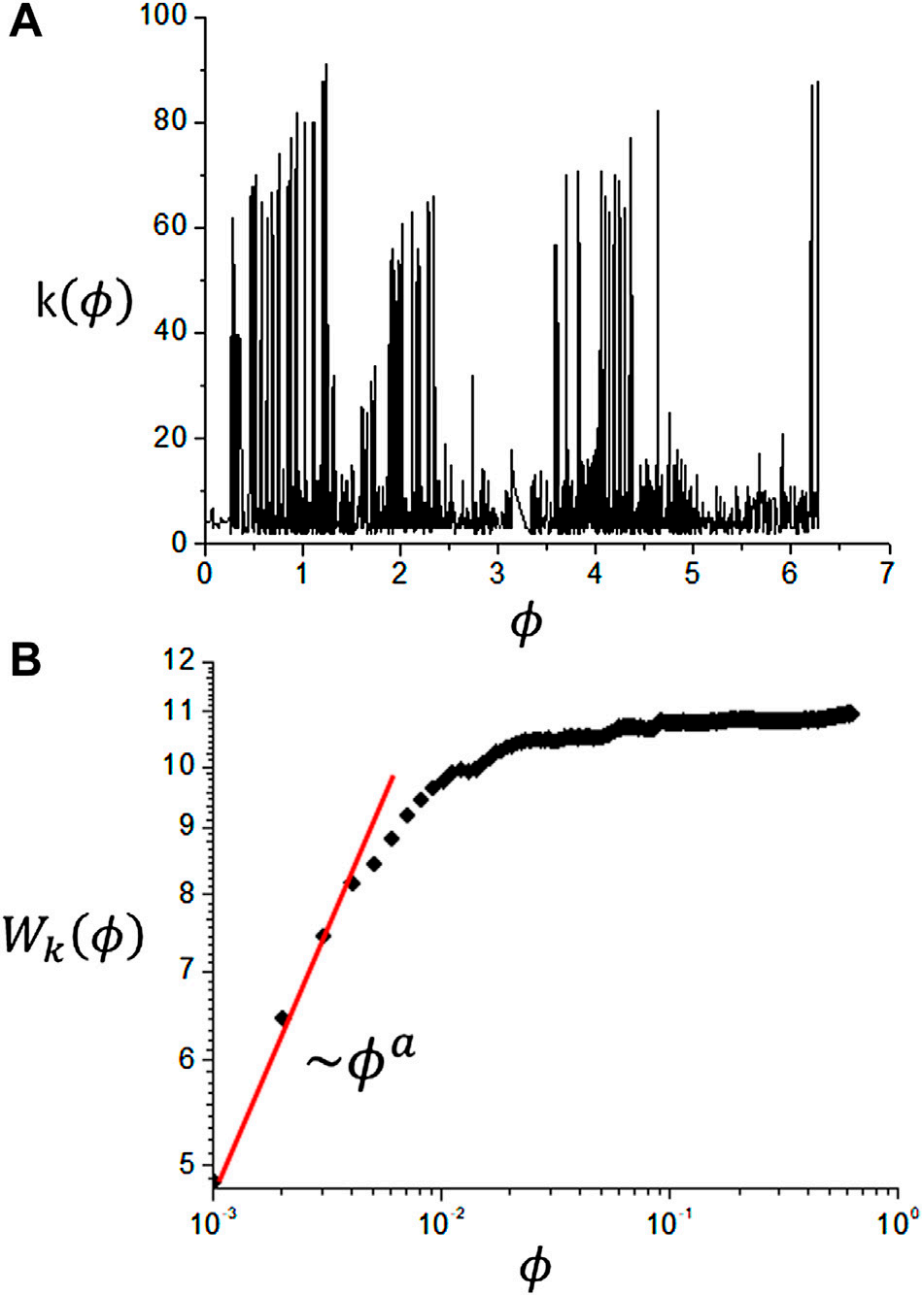
Analysis of angular ordered series **(A)** Connectivity series. **(B)** local standard deviation of the connectivity series showing a power-law dependence on *ϕ*.

It exhibits a power-law behaviour for small *ϕ* (Brú et al., 2014),

$$W_{k}∼\phi^{a}$$ (20)

where *a* represents the local variance exponent and for *ϕ* = 2*π*, *W*_*k*_ is related to the global variance or heterogeneity of the associated visibility graph, as shown in Figure 4.

#### 2.3.5 Multifractal Analysis

In order to determine the multifractal scaling exponents for the one-dimensional ordered series extracted from the tumor interface, a general procedure of fluctuation analysis is used (Kantelhardt et al., 2002) (Lopes and Betrouni, 2009). First, the profile of the ordered series is determined by a cumulative sum,

$$R\left(i\right)\equiv \overset{i}{\underset{k=1}{\sum }}\left(r_{k}-\left\langle r\right\rangle \right)$$ (21)

where ⟨*r*⟩ represents the mean radius of the tumor interface. The profile series is then partitioned into *N*_*s*_ ≡int (*N*/*s*) segments of equal length *s*, the box probability *p*_*s*_ (*v*), which is the sum of the values *r*_*k*_ within each segment *v* of size *s*, is defined as,

$$p_{s}\left(v\right)\equiv R\left(vs\right)-R\left(\left(v-1\right)s\right)$$ (22)

The scaling properties and exponents can be obtained through the partition function,

$$Z_{q}\left(s\right)\equiv \overset{N_{s}}{\underset{v=1}{\sum }}|p_{s}\left(v\right){|}^{q}$$ (23)

For large values of *s*, a power law behavior is obtained for *Z*_*q*_ (*s*), allowing for a definition of the scaling exponent *τ*_1_ (*q*) that characterizes the one-dimensional fluctuations,

$$Z_{q}\left(s\right)∼s^{\tau_{1}\left(q\right)}$$ (24)

The generalized fractal dimensions are then defined as,

$$D_{1}\left(q\right)\equiv \frac{\tau_{1}\left(q\right)}{q-1}$$ (25)

and the generalized Hurst exponents can be obtained from the relation,

$$\tau_{1}\left(q\right)=qh_{1}\left(q\right)-1$$ (26)

The ordered series that can be extracted from any slice represent a one dimensional sampling of the tumor interface and as a consequence an incomplete picture of the tumor interface fluctuations. A more general approach is possible if a two dimensional detrended fluctuation analysis (Gu and Zhou, 2006) is performed. In this case, the tumor interface is parameterized as a two dimensional array with elements *r* (*n*_*ϕ*_, *n*_*Z*_), the radii of the tumor interface, and is partitioned in two dimensional segments of size *s*. The detrended fluctuation in each segment is given by,

$$F^{2}\left(v,w,s\right)=\frac{1}{s^{2}}\overset{s}{\underset{i=1}{\sum }}\overset{s}{\underset{j=1}{\sum }}\epsilon_{v,w}^{2}\left(i,j\right)$$ (27)

where *ϵ*_*v*,*w*_ (*i*, *j*) is the difference between the cumulative sum of *r* (*i*, *j*) and its trend over the segment (*v*, *w*). The average of the detrended fluctuation over all the segments is,

$$F_{q}\left(s\right)={\left\{\frac{1}{M_{s}N_{s}}\overset{M_{s}}{\underset{v=1}{\sum }}\overset{N_{s}}{\underset{w=1}{\sum }}{\left[F\left(v,w,s\right)\right]}^{q}\right\}}^{\frac{1}{q}}$$ (28)

for *q* ≠ 0 and for *q* = 0,

$$F_{0}\left(s\right)=\exp\left\{\frac{1}{M_{s}N_{s}}\overset{M_{s}}{\underset{v=1}{\sum }}\overset{N_{s}}{\underset{w=1}{\sum }}\ln\left[F\left(v,w,s\right)\right]\right\}$$ (29)

For large values of *s*, *F*_*q*_ behaves as a power law,

$$F_{q}\left(s\right)∼s^{h_{2}\left(q\right)}$$ (30)

The multifractal nature of the fluctuation is characterized by the scaling exponents *τ*_2_ (*q*) and related to the exponents *h*_2_ (*q*) by,

$$\tau_{2}\left(q\right)=qh_{2}\left(q\right)-2$$ (31)

and Equation 25 holds for the generalized fractal dimensions *D*_2_ (*q*).

## 3 Results and Discussion

A total of 609 tumor interfaces were analyzed, discriminated as follows, 176 benign tumors including 99 meningiomas and 77 acoustic schwannomas, all of them coming from local databases, 46 Grade II and Grade III astrocytomas and oligodendrogliomas (Pedano et al., 2020) (Scarpace et al., 2019) and 387 glioblastoma multiforme Grade IV tumors (Scarpace et al., 2016) (Baid et al., 2021). The tumor interfaces were selected by its size, i.e., the number of points in the tumor interface must be greater than a certain threshold, allowing for adequate statistics in the evaluation of morphological parameters such as fractal dimension *d*_*F*_ or local roughness exponent, *α*_*loc*_. Also, the distribution of points must be as isotropic as possible, with values of the fractional anisotropy, equation (Işın et al., 2016), closest to zero. This is performed by the procedure described in Section 2.3.3 and shown in Figure 3.

### 3.1 Scaling Analysis Results

**FIGURE 5:**
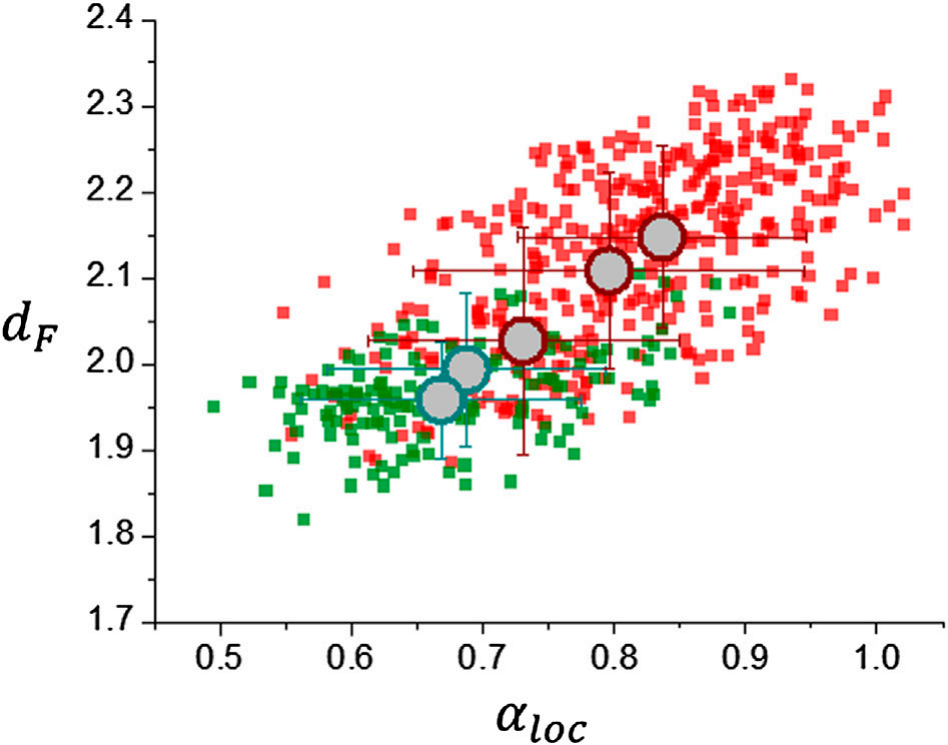
Scaling analysis results for different brain tumor databases: Glioblastoma multiforme:TCGA-GBM and BraTS 2021, and Glioma: TCGA-LGG and REMBRANDT, are represented in red color; Meningioma and Acoustic Schwannoma correspond to local databases, represented in green color. Large circles indicate average values of data points.

**FIGURE 6:**
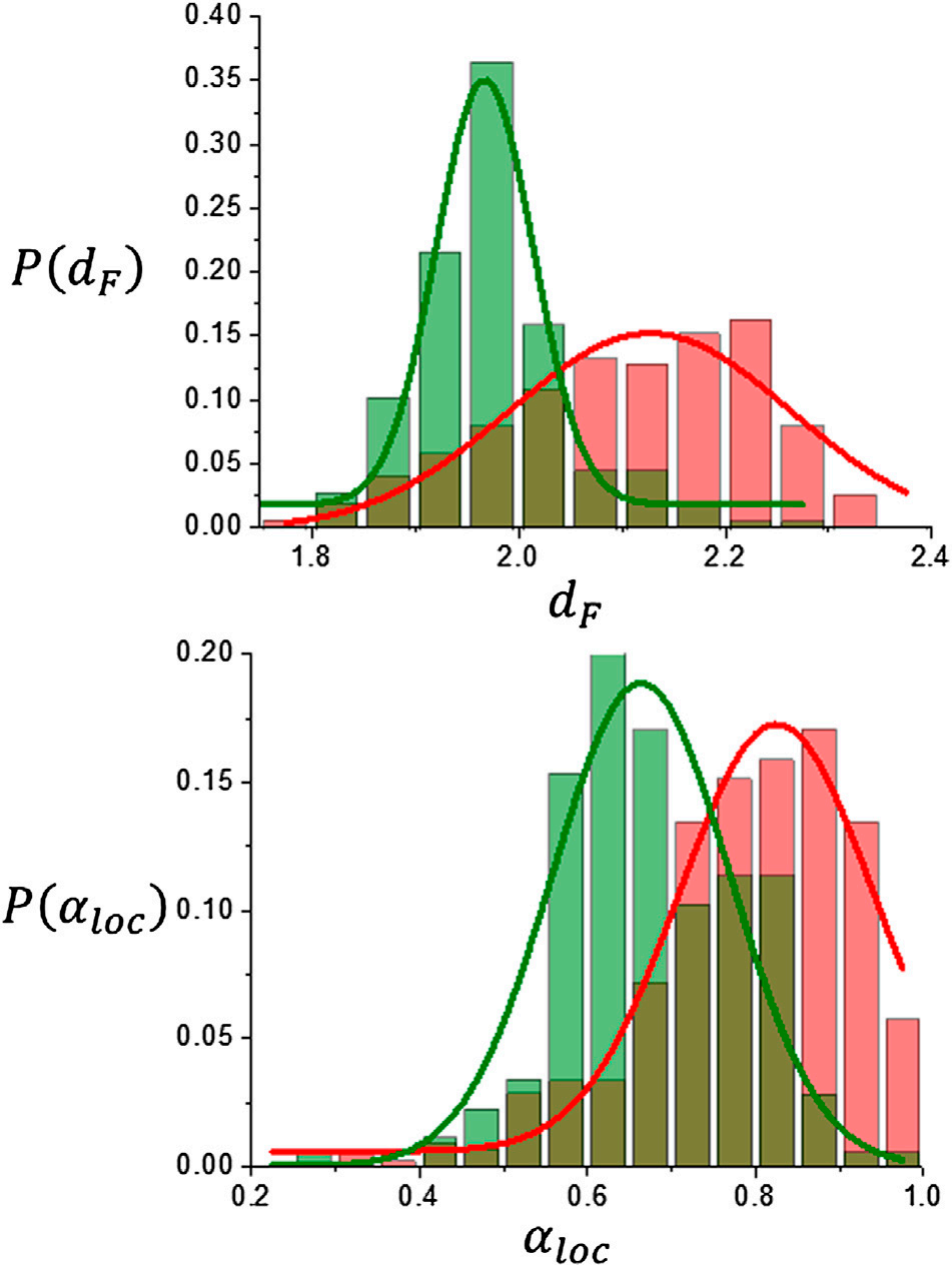
Scaling analysis results for brain tumors. Distribution histograms for *d*_*F*_ and *α*_*loc*_.

**FIGURE 7:**
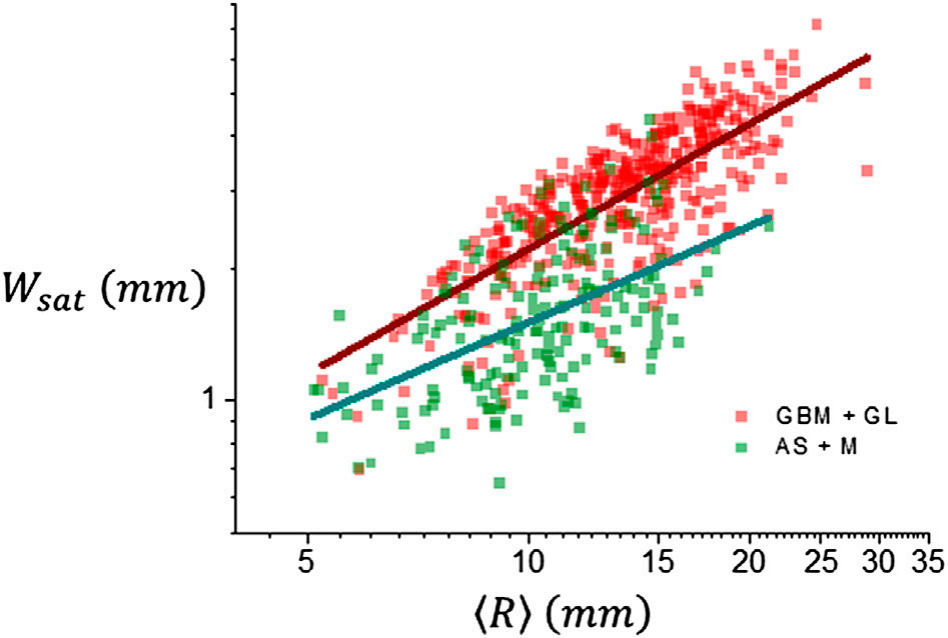
Scaling behavior of *W*_*sat*_ with tumor size *R*, according to Equation 9. Lines represent the trend of the data Values of the global roughness exponent *α* are 0.74 ± 0.09 for meningiomas and acoustic schwannomas (AS + M) and 0.94 ± 0.04 for high grade gliomas and glioblastoma multiforme (GBM + GL).

Results are shown in Figure 5 which exhibits a high dispersion of the data. Average values are indicated by large circles and correspond from left to right to meningioma, acoustic schwannoma, Grade II and Grade III glioma TCGA-LGG and REMBRANDT databases), glioblastoma multiforme and high grade glioma (BraTS 2021 database) and glioblastoma multiforme (TCGA-GBM database). These average values are summarized in Table 1. There is a clear difference between meningiomas and acoustic schwannomas, top of Table 1, with average parameters *d*_*F*_ = 1.97 ± 0.08 and *α*_*loc*_ = 0.68 ± 0.11 and gliomas, bottom of Table 1, with *d*_*F*_ = 2.11 ± 0.12 and *α*_*loc*_ = 0.80 ± 0.14, a result that is consistent with previous research using segmentation schemes based on k-means classification alone and smaller data sets (Torres Hoyos and Martín-Landrove, 2012) (Martín-Landrove et al., 2020) (Martín-Landrove et al., 2016). This fact is also shown in Figure 6 in the comparison of the corresponding histograms for *d*_*F*_ and *α*_*loc*_. The values of the local roughness exponent *α*_*loc*_ and the fractal dimension *d*_*F*_ give important information about what proliferative-invasive process is taking place in the dynamics of tumor growth, if the sum of these parameters is close to the Euclidean dimension, *d*_*E*_, as reflected by Equation 8, the tumor growth dynamics corresponds to a ballistic growth model, characterized by the ansatz of Family-Vicsek (Family and Vicsek, 1991) (Barabasi and Stanley, 1995). Inspection of Table 1 reveals that this is indeed the case for Grade II and III gliomas and glioblastoma multiforme, *d*_*F*_ + *α*_*loc*_ = 2.92 ± 0.22. In the case of meningiomas and acoustic schwannomas, the tumor growth dynamics corresponds to a different growth model since *d*_*F*_ + *α*_*loc*_ = 2.65 ± 0.16. Also, from Table 1, lacunarity values, *λ*, exhibits a variation that it is in correspondence with fractal dimension, *d*_*F*_. Another important feature comes from the results shown in Figure 7 which depicts the dependence of *W*_*sat*_ with respect to the average size of the tumor lesion, ⟨*R*⟩. Trend of the data conforms to a power law according to Equation 9 and the results are summarized in Table 2. There is a clear difference between tumor groups, for gliomas, *α* = 0.948 ± 0.038, and for meningiomas and acoustic schwannomas, *α* = 0.730 ± 0.087, which is what has to be expected if the growth dynamics governing the tumor interface corresponds to a more invasive process as it happens to be the case for malignant tumors.

**TABLE 1:** Scaling analysis results for dynamical parameters.

Tumor type	*d*_*F*_	*α*_*loc*_	*d*_*F*_ + *α*_*loc*_	*λ*
Acoustic Schwannoma	1.99 ± 0.11	0.69 ± 0.03	2.68 ± 0.18	0.46 ± 0.21
Meningioma	1.96 ± 0.11	0.67 ± 0.03	2.63 ± 0.15	0.46 ± 0.21
Grade II and Grade III Glioma*	2.03 ± 0.13	0.73 ± 0.03	2.76 ± 0.23	0.52 ± 0.25
Glioblastoma multiforme^†^	2.15 ± 0.10	0.84 ± 0.04	2.98 ± 0.18	0.50 ± 0.24
Glioblastoma multiforme^‡^	2.11 ± 0.12	0.80 ± 0.04	2.90 ± 0.23	0.49 ± 0.24

Databases are: (*) TCGA-LGG and REMBRANDT, (†) TCGA-GBM and (‡) BraTS Challenge 2021.

**TABLE 2:** Scaling analysis results for global dynamics of the tumor interface width.

Tumor type	*W*_*sat*_ (*mm*)	⟨*R*⟩ (*mm*)	*α*
Acoustic Schwannoma	1.64 ± 0.65	10.07 ± 2.48	0.73 ± 0.15
Meningioma	1.67 ± 0.65	11.19 ± 2.96	0.76 ± 0.11
Grade II and Grade III Glioma*	3.02 ± 1.23	13.21 ± 3.89	1.08 ± 0.15
Glioblastoma multiforme^†^	3.35 ± 1.00	15.08 ± 3.99	0.91 ± 0.06
Glioblastoma multiforme^‡^	2.94 ± 0.98	13.27 ± 3.70	0.93 ± 0.06

Databases are: (*) TCGA-LGG and REMBRANDT, (†) TCGA-GBM and (‡) BraTS Challenge 2021.

### 3.2 Regularity Measures Results

**FIGURE 8:**
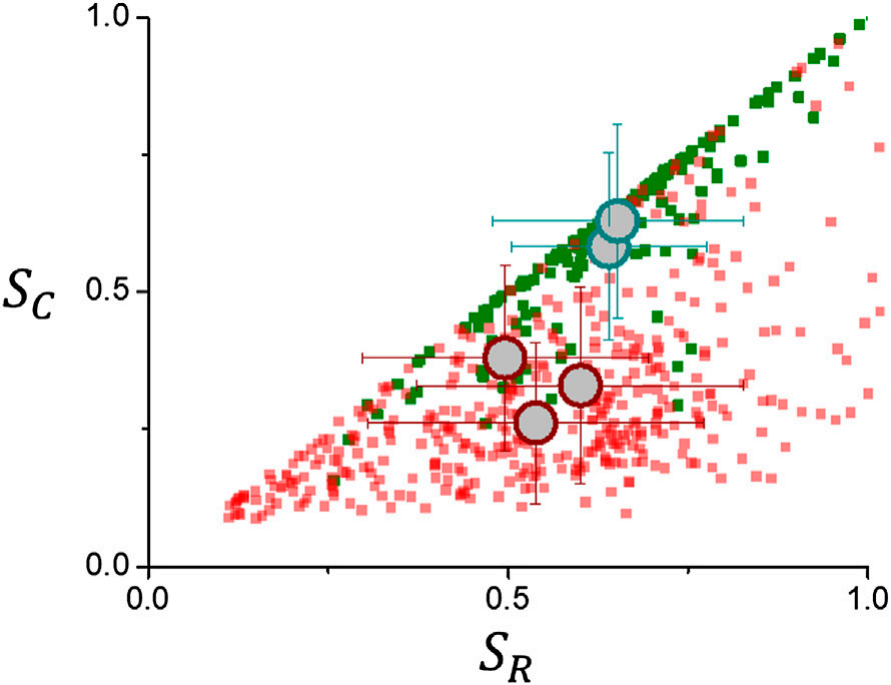
Regularity measures for brain tumors. Circles represent average values for meningiomas and acoustic schwannomas (green) and high grade gliomas and glioblastoma multiforme (red).

**FIGURE 9:**
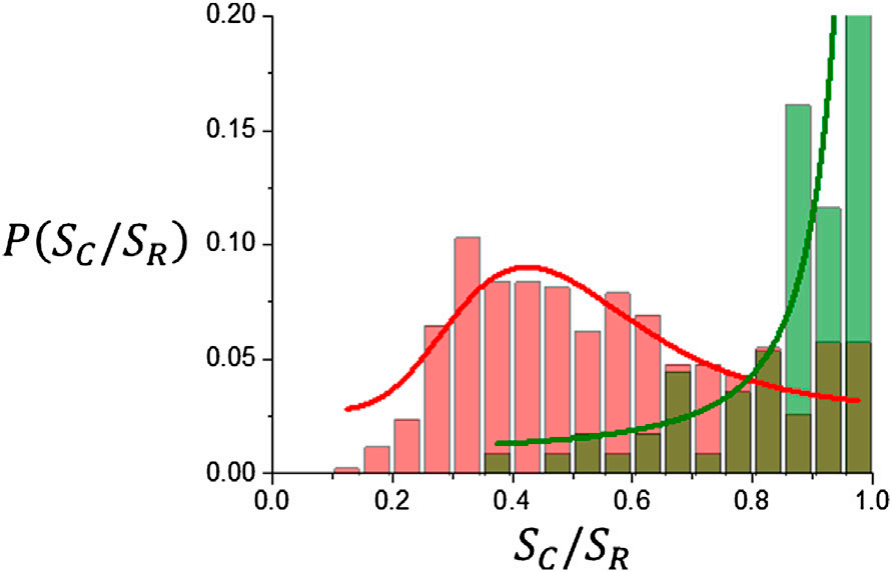
Distribution histograms for *S*_*C*_/*S*_*R*_. Meningiomas and acoustic schwannomas are indicated in green and gliomas in red.

**FIGURE 10:**
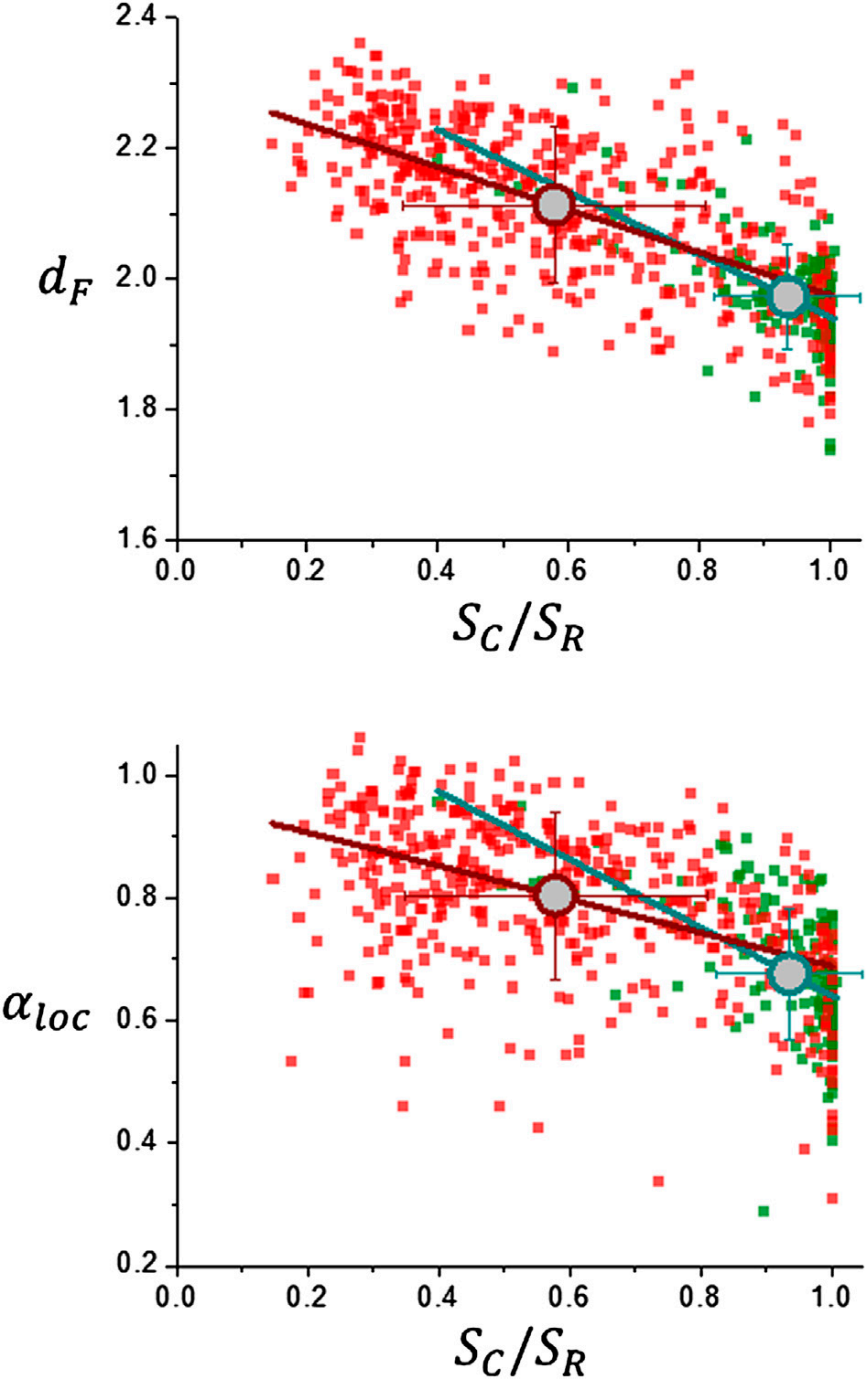
Relationship between scaling analysis parameters *d*_*F*_ and *α*_*loc*_ with regularity measure ratio *S*_*C*_/*S*_*R*_. Large circles represent average values: meningiomas and acoustic schwannomas (green) and gliomas (red).

The relationship between *S*_*C*_ and *S*_*R*_ are shown in Figure 8. Data points are dispersed below the diagonal since *S*_*C*_ takes into account both inner and outer surfaces while *S*_*R*_ only takes into account the outer surface (Pérez-Beteta et al., 2018). Data points along the diagonal correspond to tumors that lack the presence of either contrast free or necrotic volumes, a condition that occurs more frequently for meningiomas and acoustic schwannomas than for gliomas as seen in Figure 8. Large circles in Figure 8 correspond to average values that are summarized in Table 3. The average values of *S*_*R*_ for meningiomas and acoustic schwannomas, 0.64 ± 0.16 and gliomas, 0.57 ± 0.23, do not differ significantly. On the other hand, average values of *S*_*C*_ are 0.61 ± 0.17 for meningiomas and acoustic schwannomas, and 0.31 ± 0.17 for gliomas, have a difference that clearly discriminates between these groups. In order to enhance the difference among tumor groups, the ratio *S*_*C*_/*S*_*R*_ is used, as seen from Table 3. Figure 9 shows frequency distributions for meningiomas and acoustic schwannomas, with an average value ⟨*S*_*C*_/*S*_*R*_⟩ = 0.94 ± 0.11, and gliomas with ⟨*S*_*C*_/*S*_*R*_⟩ = 0.58 ± 0.23. Since regularity measures are related to the fractal properties of the tumor interface (Pérez-Beteta et al., 2018) there must be a certain correlation to the scaling parameters *d*_*F*_ and *α*_*loc*_. For tumors with *S*_*C*_/*S*_*R*_ close to 1, fractal dimension, *d*_*F*_, should be close to 2 and *α*_*loc*_ should have its lowest value, i.e., tumor surface is regular and smooth. As *S*_*C*_/*S*_*R*_ decreases it is expected an increase in the scaling parameters. Figure 10 shows this trend for the dependence of *d*_*F*_ and *α*_*loc*_ on *S*_*C*_/*S*_*R*_. The slopes that characterize the linear trend are summarized in Table 4.

**TABLE 3:** Regularity measures results for different brain tumor types.

Tumor type	*S*_*R*_	*S*_*C*_	*S*_*C*_/*S*_*R*_
Acoustic Schwannoma	0.64 ± 0.14	0.58 ± 0.17	0.90 ± 0.15
Meningioma	0.65 ± 0.17	0.63 ± 0.18	0.96 ± 0.06
Grade II and Grade III Glioma*	0.50 ± 0.20	0.38 ± 0.17	0.78 ± 0.20
Glioblastoma multiforme^†^	0.54 ± 0.23	0.26 ± 0.15	0.53 ± 0.23
Glioblastoma multiforme^‡^	0.60 ± 0.23	0.33 ± 0.18	0.56 ± 0.22

Databases are: (*) TCGA-LGG and REMBRANDT, (†) TCGA-GBM and (‡) BraTS Challenge 2021.

**TABLE 4:** Trend parameters for data points in (*d*_*F*_, *S*_*C*_/*S*_*R*_) and (*α*_*loc*_, *S*_*C*_/*S*_*R*_).

Tumor type	(*d*_*F*_, *S*_*C*_/*S*_*R*_)	(*α*_*loc*_, *S*_*C*_/*S*_*R*_)
M + AS	− 0.477 ± 0.041	− 0.552 ± 0.059
GL + GBM	− 0.327 ± 0.019	− 0.272 ± 0.025

M, Meningioma; AS, Acoustic schwannoma; GL, High grade glioma; GBM, Glioblastoma multiforme.

### 3.3 Ordered Series, Visibility Graphs and Multifractal Analysis Results

**FIGURE 11:**
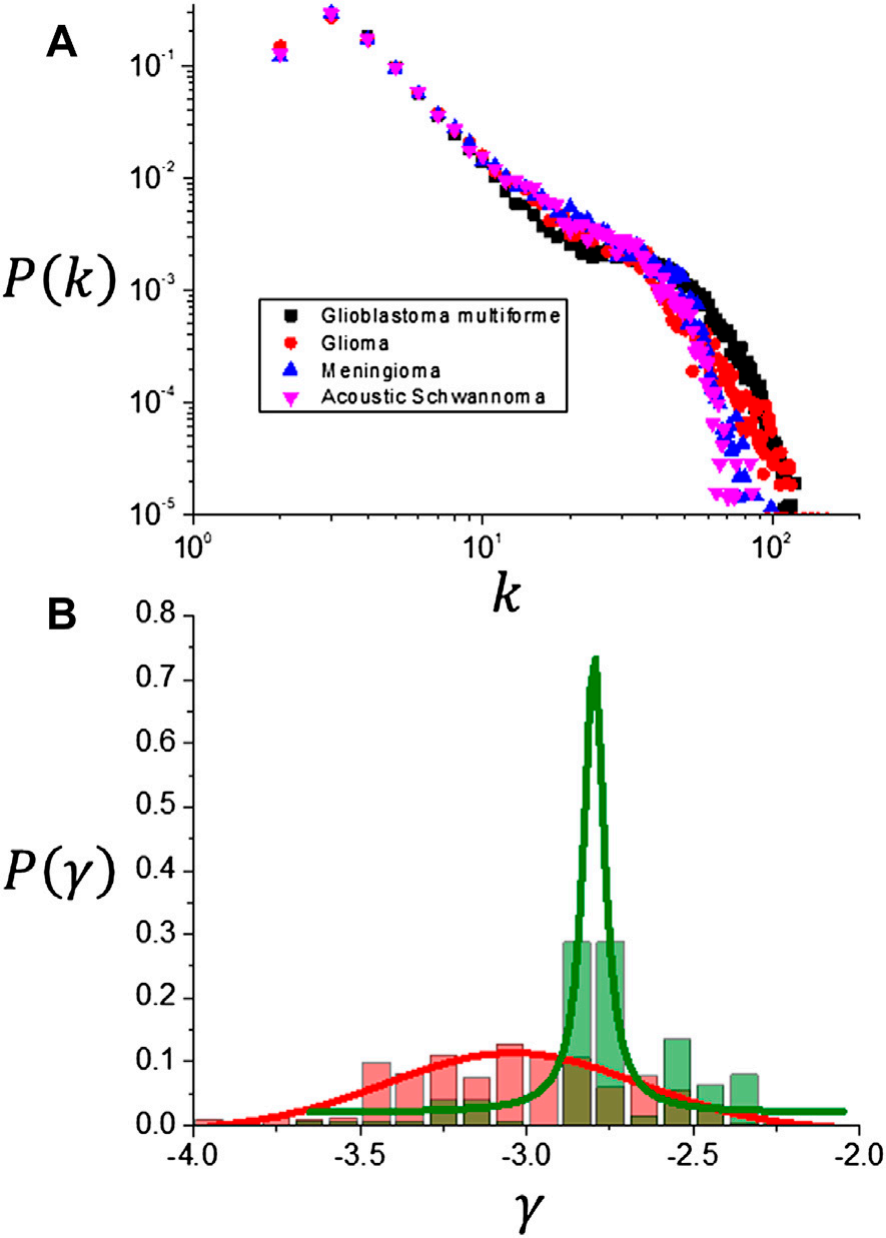
**(A)** Visibility graph degree distributions *P* (*k*) for different tumor types. A power law behavior region is observed for *k* < 20 and the probability decreases abruptly beyond that value. **(B)** Frequency distributions for the exponent *γ* of the power law behavior.

**FIGURE 12:**
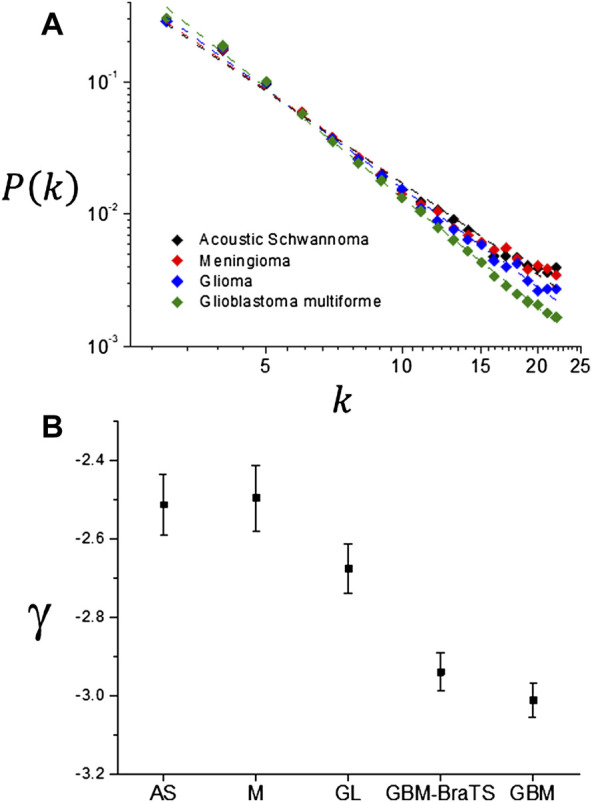
**(A)** Detail of the power law region for the visibility graph degree distributions. **(B)** Exponent *γ* dependence on tumor type.

**FIGURE 13:**
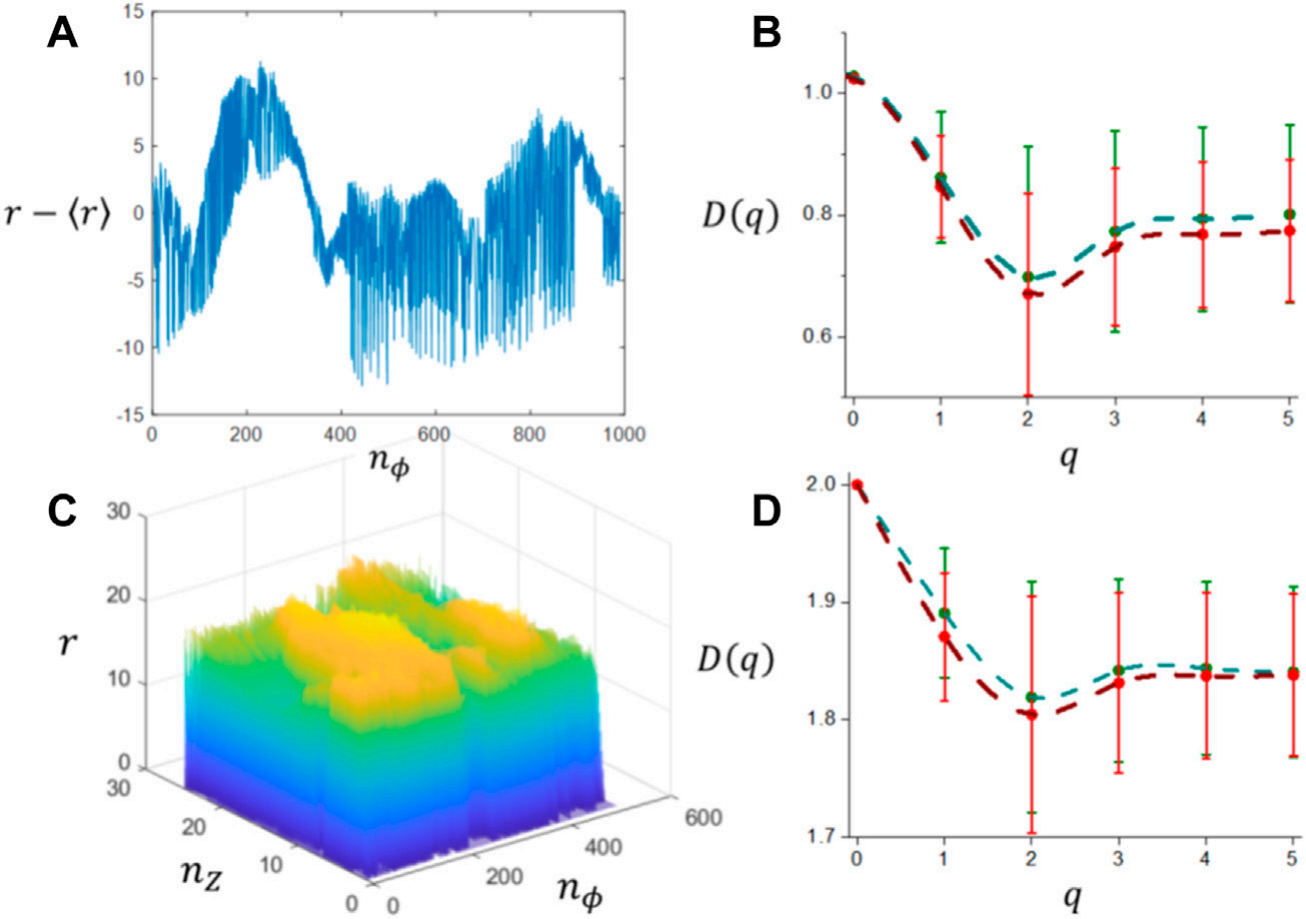
Multifractal and detrended fluctuation analysis results. **(A)** Typical ordered series extracted from an slice; **(B)** Generalized fractal dimensions associated to the ordered series obtained by multifractal analysis using the partition function *Z*; **(C)** Two dimensional landscape for *r* as a function of *n*_*Z*_ and *n*_*ϕ*_, and **(D)** Generalized fractal dimensions obtained by 2D Detrended Fluctuation Analysis. In **(B,D)**, meningiomas and acoustic schwannomas are represented in green and gliomas in red.

Ordered series were extracted from the tumor interface for those slices that contain the maximum number of interface points, visibility graphs were generated and the degree distribution functions were obtained. Results for the average of *P* (*k*) distributions obtained for each of the different tumor types are shown in Figure 11A. For values of the connectivity index *k* > 20, *P* (*k*) decays abruptly due to the fact that the ordered series has a finite size which limits the probability *P* (*k*). For values of the connectivity index *k* < 20 all distributions exhibit a power law behavior as shown in Figure 12A with slopes that are summarized in Table 5 and shown in Figure 12B. If the tumor types are discriminated only into two classes: one including meningiomas and acoustic schwannomas and the other including high grade gliomas and glioblastoma multiforme, the slope distributions are clearly distinct for each tumor class as shown in Figure 11B. This result suggest the possibility of using *γ* as a possible parameter that characterizes tumor interface dynamics supported by the result shown in Figure 12B. Multifractal analysis results are summarized in Figure 13. As expected, the evaluation of generalized fractal dimensions for the one dimensional sampling of the tumor interface, Figure 13A, does not provide with enough information to discriminate between these two classes as seen in Figure 13B and summarized in Table 6. On the other hand, two dimensional detrended fluctuation analysis of the tumor interface yield some differences in the average values of *D* (*q*), as shown in Table 6 and Figure 13D, that possibly could be improved by an adequate sampling of the tumor interface, i.e., higher image longitudinal and transverse resolution. In any case, generalized fractal dimensions do not discriminate appropriately between tumor types.

**TABLE 5:** Visibility graphs results for different brain tumor types.

Tumor type	*λ*	*a*
Acoustic Schwannoma	− 2.513 ± 0.078	0.44 ± 0.18
Meningioma	− 2.496 ± 0.083	0.51 ± 0.34
Grade II and Grade III Glioma*	− 2.676 ± 0.063	0.56 ± 0.36
Glioblastoma multiforme^†^	− 3.012 ± 0.043	0.47 ± 0.14
Glioblastoma multiforme^‡^	− 2.940 ± 0.049	0.49 ± 0.21

Databases are: (*) TCGA-LGG and REMBRANDT, (†) TCGA-GBM and (‡) BraTS Challenge 2021.

**TABLE 6:** Generalized fractal dimensions obtained by Detrended Fluctuation Analysis on one dimensional ordered series, *D*_1_ (1) and *D*_1_ (2), and two dimensional interface data space *r* (*n*_*ϕ*_, *n*_*Z*_).

Tumor type	*D*_1_ (1)	*D*_1_ (2)	*D*_2_ (1)	*D*_2_ (2)
Acoustic Schwannoma	0.88 ± 0.08	0.72 ± 0.16	1.88 ± 0.04	1.80 ± 0.08
Meningioma	0.85 ± 0.12	0.68 ± 0.25	1.90 ± 0.06	1.83 ± 0.11
Grade II and Grade III Glioma*	0.83 ± 0.09	0.63 ± 0.18	1.87 ± 0.04	1.80 ± 0.09
Glioblastoma multiforme^†^	0.85 ± 0.09	0.67 ± 0.17	1.86 ± 0.05	1.79 ± 0.09
Glioblastoma multiforme^‡^	0.85 ± 0.08	0.68 ± 0.16	1.88 ± 0.06	1.81 ± 0.05

Databases are: (*) TCGA-LGG and REMBRANDT, (†) TCGA-GBM and (‡) BraTS Challenge 2021.

## 4 Conclusion

**FIGURE 14:**
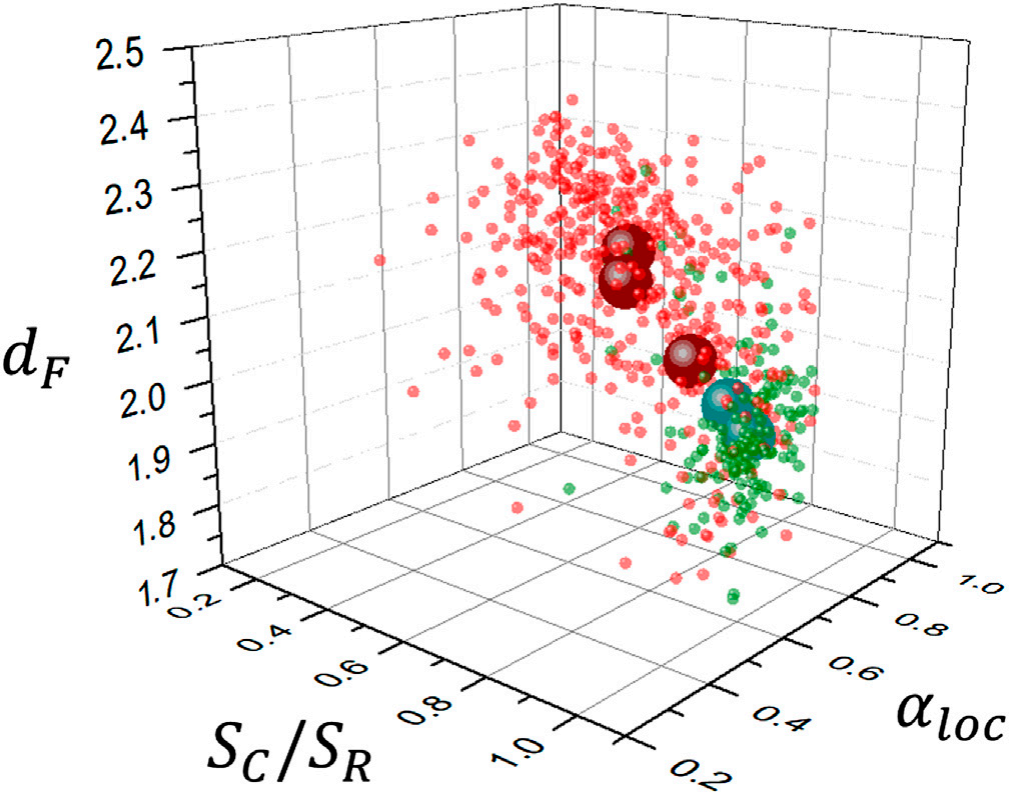
Relevant morphological and scaling parameters used to discriminate between meningiomas and acoustic schwannomas (green) and gliomas (red). Average values are represented as large dots.

A method based on dynamic quantum clustering is used to perform contrast enhanced MRI of brain tumors. Tumor interfaces can be classified according to scaling analysis parameters such as the fractal dimension, *d*_*F*_ and the local roughness exponent, *α*_*loc*_ which clearly differentiate between the growth dynamics of different tumor types adding support of a ballistic growth model for gliomas and glioblastomas, following the Family-Vicsek ansatz and a non-ballistic growth model for other neoplasias such as meningiomas and acoustic schwannomas. Among the regularity measures, the ratio *S*_*C*_/*S*_*R*_ exhibit some correlation with the scaling parameters and clearly discriminates between gliomas and meningiomas or acoustic schwannomas. The relation between *d*_*F*_, *α*_*loc*_ and *S*_*C*_/*S*_*R*_ is shown in Figure 14. Parameters obtained in series extracted from the tumor interface are size sensitive but nevertheless exhibit differences that could be used for tumor classification and in particular its growth dynamics, through the exponent *γ*. Generalized fractal dimensions obtained by two dimensional detrended fluctuation analysis could possibly give significant differences if the tumor interface could be sampled with higher resolution. Further research should take into account combination of different MRI modalities.

## Data Availability

The raw data supporting the conclusions of this article will be made available by the authors, without undue reservation.
